# A Multimodal Ultrasound Approach Combining Transperineal and Transrectal Shear Wave Elastography for Early Prediction of Stress Urinary Incontinence in Women

**DOI:** 10.1002/jum.70099

**Published:** 2025-11-10

**Authors:** Yidan Wang, Jing Feng, Jingyan Xie, Yang Yang, Yaping Wang, Yujuan Li, Jiajun Xu

**Affiliations:** ^1^ Department of Ultrasonography Nanjing First Hospital, Nanjing Medical University Nanjing People's Republic of China; ^2^ Department of Gynecology Nanjing First Hospital, Nanjing Medical University Nanjing People's Republic of China; ^3^ Department of Obstetrics Nanjing First Hospital, Nanjing Medical University Nanjing People's Republic of China

**Keywords:** shear wave elastography, SUI, transperineal ultrasound, urethral stiffness

## Abstract

**Objectives:**

This study aims to evaluate the diagnostic value of a novel multimodal approach combining transperineal ultrasound (TPUS), transrectal dual‐plane ultrasound, and shear wave elastography (SWE) in predicting stress urinary incontinence (SUI).

**Methods:**

A total of 70 women diagnosed with SUI and 110 healthy controls were included. Clinical data such as age, body mass index (BMI), mode of delivery, and relevant medical history were collected. Pelvic floor ultrasound was performed using TPUS and transrectal dual‐plane ultrasound. Key parameters, including bladder neck mobility, urethral rotation angle, urethral length, and urethral stiffness measured by SWE, were recorded and analyzed. Statistical analysis was conducted using SPSS version 27.0, and a multifactorial predictive model was developed.

**Results:**

Significant differences were observed between the SUI and control groups in bladder neck mobility (*p* = .034), urethral rotation angle (*p* = .059), and urethral stiffness measured by SWE (*p* < .001). The average Young's modulus of the urethral sphincter was significantly lower in the SUI group (39.22 ± 5.83 kPa) compared to the control group (52.11 ± 9.24 kPa). Age and average urethral sphincter elasticity were identified as independent risk factors for SUI. The multifactorial model demonstrated high clinical applicability with an AUC of 0.891 (sensitivity: 84%, specificity: 80%).

**Conclusions:**

The combination of TPUS, transrectal dual‐plane ultrasound, and SWE provides a reliable, non‐invasive diagnostic tool for predicting SUI. The study highlights the importance of urethral stiffness and bladder neck mobility in the pathophysiology of SUI. This multi‐modal approach shows strong potential for early detection and personalized treatment strategies. This multimodal approach offers a non‐invasive, reliable tool for early SUI detection and personalized management.

**Abbreviations**
AUC: area under the curve
BMI: body mass index
CST: cough stress test
EMG: electromyography
ICS: International Continence Society
PFMT: pelvic floor muscle training
ROC: receiver operating characteristic
ROI: region of interest
SUI: stress urinary incontinence
SWE: shear wave elastography
TPUS: transperineal ultrasound
UTI: urinary tract infection

Stress urinary incontinence (SUI) is a common and debilitating condition, characterized by the involuntary leakage of urine during physical activities that increase intra‐abdominal pressure, such as coughing, sneezing, lifting heavy objects, or exercising. SUI predominantly affects women, especially those who have had multiple pregnancies, experienced childbirth, or are in advanced age.^1^ The condition significantly impacts quality of life, leading to emotional distress, social embarrassment, and limitations in daily activities. As one of the most common forms of urinary incontinence, the prevalence of SUI varies across different populations.^2^, ^3^ According to the International Continence Society (ICS), approximately 25–45% of women worldwide suffer from some form of urinary incontinence, with SUI being the most common subtype.^3^ Its prevalence increases with age, being more pronounced in postmenopausal women, particularly those who have had vaginal deliveries. With aging populations and lifestyle changes, the global burden of SUI is expected to rise.

SUI diagnosis traditionally hinges on subjective symptom reporting (eg, validated questionnaires) and invasive urodynamic testing, which measures bladder pressure and urethral function during filling and voiding.^4^, ^5^ Although urodynamics remains the diagnostic gold standard,^5^ its clinical utility is constrained by high costs, patient discomfort, and limited availability—particularly in resource‐limited settings.^6^ Crucially, even urodynamic metrics fail to elucidate the underlying structural and functional abnormalities driving SUI, such as urethral sphincter degeneration or pelvic floor laxity.^7^ This knowledge gap impedes personalized treatment strategies, as clinicians lack tools to differentiate between anatomy‐driven and function‐dominant SUI subtypes. To address these gaps, this study introduces a novel multimodal ultrasound protocol integrating transperineal ultrasound (TPUS), transrectal dual‐plane imaging, and shear wave elastography (SWE). This approach uniquely combines anatomical and functional assessments to comprehensively evaluate SUI risk factors.

Recent advancements in ultrasound technology, particularly pelvic floor ultrasonography, have enabled a more comprehensive assessment of SUI. Ultrasound is non‐invasive, cost‐effective, and widely accessible, making it an attractive tool for evaluating the bladder, urethra, and pelvic floor muscles in SUI patients. Several studies^8^, ^9^ have explored the use of TPUS to assess key parameters such as bladder neck mobility, urethral rotation angle, and bladder posterior angle—factors known to correlate with the severity of SUI. Previous studies on TPUS focused on anatomical parameters (eg, bladder neck mobility), yet failed to assess functional properties of the urethral sphincter.

In this context, SWE has emerged as an innovative ultrasound technique for assessing tissue stiffness and elasticity.^10^ SWE measures tissue stiffness by quantifying its elastic properties, which can provide important information on the functional status of the urethral sphincter and pelvic floor muscles. This technique holds considerable promise as a diagnostic tool for assessing urethral function in SUI patients, although its application in pelvic floor dysfunction remains underexplored. SWE provides a quantitative measure of tissue stiffness, which may reflect the functional integrity of the urethral sphincter—a critical yet underexplored aspect in SUI pathogenesis.

To further enhance the diagnostic capacity of ultrasound for SUI, this study aims to combine TPUS with transrectal dual‐plane ultrasound to provide a more comprehensive evaluation of bladder function and urethral characteristics. In addition to traditional measurements, such as bladder neck mobility and urethral rotation angle, this study will also incorporate novel assessments of urethral length and muscle thickness. By integrating SWE to measure urethral elasticity, we aim to gain a more holistic understanding of the bladder's ability to maintain continence. This study aims to develop a multimodal ultrasound protocol integrating TPUS, transrectal dual‐plane imaging, and SWE to comprehensively evaluate both anatomical and functional predictors of SUI. This exploratory study focuses on non‐invasive ultrasound parameters as potential surrogates for functional metrics. While urodynamics remain the gold standard, our approach aims to identify complementary biomarkers for future validation in urodynamically characterized cohorts.

## Materials and Methods

### Study Subjects

This study is a cross‐sectional, retrospective analysis of pelvic floor ultrasound data and clinical information from female patients who visited the Department of Obstetrics and Gynecology at the Nanjing First Hospital between January 2023 and August 2024. Sample size was calculated using GPower 3.1, assuming an effect size (Cohen's d) of 0.5 for urethral stiffness between groups, with *α* = 0.05 and 80% power. This yielded a minimum of 60 participants per group; we enrolled 70 SUI patients and 110 controls to account for potential exclusions. The study included 70 women diagnosed with SUI, defined by involuntary urine leakage following physical activities such as coughing, sneezing, or other actions that increase intra‐abdominal pressure, or those with a positive result on the cough stress test (CST). The control group consisted of 110 healthy women. Ethical approval for this study was granted by the Human Research Ethics Committee.

Inclusion criteria for the SUI group:

1. Women experiencing uncontrollable urine leakage following physical activities that increase intra‐abdominal pressure, such as coughing, sneezing, or running.
2. Positive CST result.

Exclusion criteria:

1. History of pelvic or pelvic floor surgery or physiotherapeutic interventions.
2. Individuals with active urinary tract infection (UTI) defined by positive urine culture or symptoms (dysuria, urgency).
3. Individuals with urinary retention or voiding difficulties.
4. Individuals with urinary tract tumors.
5. History of pelvic radiation therapy.
6. Incomplete ultrasound or clinical data.

### Instruments and Methods

**Figure 1:**
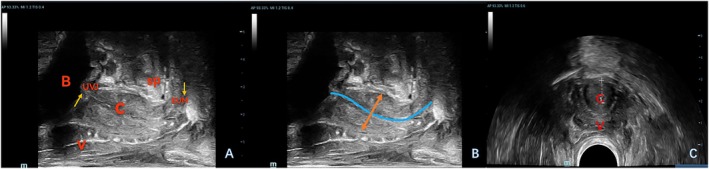
The transrectal linear array probe is used to observe and measure the urethra and its surrounding structures. **A** shows the 2‐dimensional image of the urethra and its surrounding structures and **B** shows the measurement method (measure the width of the broadest spindle‐shaped structure in the middle and upper segments of the urethral sphincter, as well as the distances between the mucosal layers of the anterior and posterior urethral walls on both sides). **C**, The convex array probe is used to observe the cross‐sectional structure of the urethra B, urethra; V, vagina; C, urethra; UVJ, urethrovesical junction; EUM, external urethral meatus.

**Figure 2:**
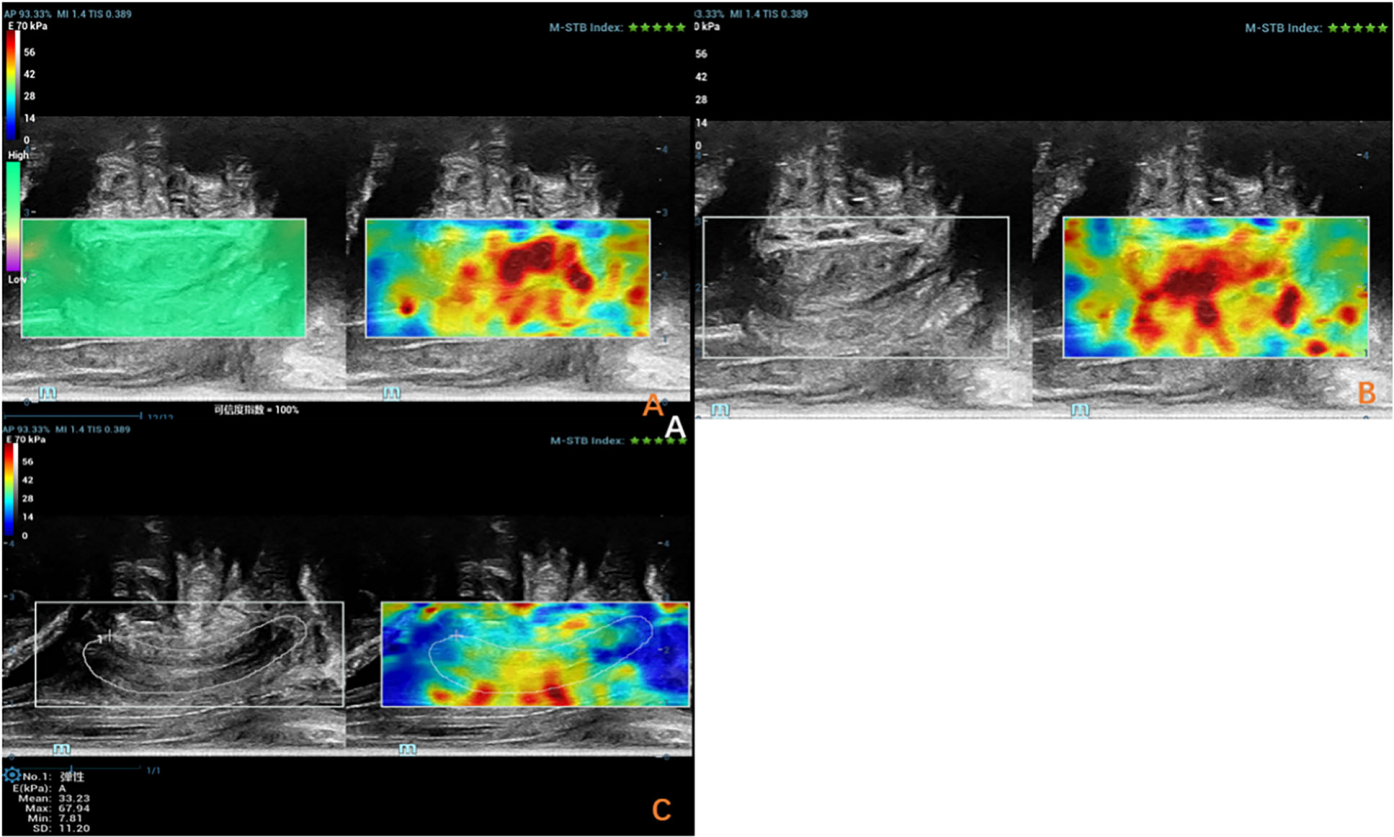
SWE was performed on the urethral sphincter. Image **A** indicates that the image quality is stable, with a confidence interval of 100% and an M‐STB index of 5 stars. Image **B** shows that the middle segment of the urethral sphincter appears red, with a relatively high Young's modulus value. Image **C**, a custom‐defined region of interest (ROI) is used to enclose the entire urethral sphincter to obtain the stiffness of the urethral sphincter in this area, as well as the maximum elasticity (SWE Max), the minimum elasticity (SWE Min).

Pelvic floor ultrasound examinations were conducted using the Mindray Resona R9 ultrasound system (Mindray Bio‐Medical Electronics Co., China) equipped with a 3D volumetric probe (1.3–8.2 MHz), a dual‐plane intracavitary probe (4.8–11.0 MHz), and a convex array probe (3.5–9.5 MHz). Patients were positioned in the lithotomy posture for bladder imaging. Initially, the volume probe was placed in the perineum in the sagittal plane to obtain dynamic images of the median sagittal section of the pelvic floor in both resting and Valsalva states. Relevant indicators, including the area of the levator ani hiatus during maximum Valsalva maneuver, were recorded. Following this, the biplanar probe was inserted into the rectum. The transverse section of the periurethral sphincter was imaged using the convex array probe, and the linear array probe was employed to measure the urethral length and periurethral sphincter thickness (Figure 1). Real‐time SWE was then activated. The sampling frame was set to encompass the entire urethra (with cotton swabs used to indicate any unclear external urethral boundary), ensuring that the urethra was centered within the image, and the image remained stable (confidence interval >95%, with 5‐star green quality). Images were captured, and the SWE Young's modulus of the urethra was measured by manually delineating a custom region of interest (ROI) around the entire urethra (Figure 2). The maximum, average, and minimum values of *Young's modulus* within the ROI were then recorded. To ensure measurement reproducibility, a single experienced sonographer conducted 3 consecutive SWE assessments. All procedures adhered to the guidelines set forth by the International Urogynecological Association, and all ultrasound examinations were performed by a single pelvic floor ultrasound specialist. Shear wave velocity serves as a quantitative index for tissue stiffness, with a higher Young's modulus correlating with increased tissue hardness.

### Data Collection

Clinical data were collected for all subjects, including age, body mass index (BMI), mode of delivery, frequency of deliveries, and relevant medical history. Ultrasound data included bladder neck mobility, urethral rotation angle, bladder posterior angle, levator ani hiatus area at maximum Valsalva maneuver, diameters of the levator hiatus (upper and lower), urethral length, urethral sphincter thickness, and SWE measurements of the urethral sphincter Young's modulus.

### Statistical Analysis

Statistical analysis was performed using SPSS version 27.0 (IBM). Continuous variables were expressed as mean ± standard deviation (x̄ ± s), and differences between groups were analyzed using independent sample *t*‐tests. Categorical data were presented as frequency or percentage, and comparisons between groups were conducted using the χ^2^ test. Binary logistic regression analysis and Spearman correlation analysis were employed to explore the relationship between the measured variables and the occurrence of SUI. Multivariate regression analysis was performed to identify the factors influencing the prediction of SUI. Receiver operating characteristic (ROC) curves were generated to evaluate the co‐predictive value of age and mean urethral shear wave elasticity for SUI. The ROC curves were further used to assess the predictive performance of the model. Variables with *p* < .1 in univariate analysis were included in the multivariate logistic regression model. To validate the model's generalizability across different datasets, 10‐fold cross‐validation was performed to assess model generalizability, with 90% of data randomly selected for training and 10% for validation in each fold. A *p*‐value of <.05 was considered statistically significant.

## Result

We analyzed data from 179 individuals, including 70 patients with SUI and 109 healthy controls. Significant differences were observed between the 2 groups in terms of age, BMI, and mode of delivery. The mean age of the SUI group was significantly higher (53.51 ± 13.44 years) compared to the control group (43.35 ± 17.49 years) (*p* < .001). In the SUI group, 90% of the patients (63/70) had delivered vaginally, whereas only 68.8% (75/109) of the control group had a vaginal delivery, a notable difference. These findings align with known risk factors for SUI. No significant differences were observed in BMI (*p* = .17) or parity, indicating that their role in SUI under the conditions of our study is limited (Table 1).

**Table 1:** Demographics of the SUI and Control Groups

Characteristic	SUI (n = 70)	Control (n = 109)	*p* Value
Age (year)	53.51 ± 13.44	43.35 ± 17.49	<.001
BMI (kg/m^2^)	23.88 ± 3.75	22.69 ± 2.81	.17
Parity (N)			
N = 1	47	75	.469
N = 2	17	26	
N = 3	3	7	
N = 4	3	1	
Vaginal delivery	63	75	.001

Among the pelvic floor ultrasound parameters, bladder neck mobility and bladder posterior angle were significantly greater in the SUI group than in the control group (*p* = .034 and *p* = .059, respectively). The urethral rotation angle was also significantly higher in the SUI group (65.03 ± 42.87°) compared to the control group (53.85 ± 35.32°). However, no significant differences were found in the anal levator hiatus area, anteroposterior diameter, transverse diameter, urethral length, or urethral sphincter thickness between the 2 groups (Table 2).

**Table 2:** Differences Between the SUI and Control Groups

Characteristic	SUI (n = 70)	Control (n = 109)	*p* Value
	Mean ± SD	Mean ± SD	
BND (mm)	2.45 ± 1.09	1.91 ± 1.09	.002
URA (°)	144.55 ± 21.68	136.7 ± 25.61	.034
RVA (°)	65.03 ± 42.87	53.85 ± 35.32	.059
Area of the LH	22.53 ± 6.18	20.97 ± 7.42	.136
Anteroposterior diameter of the LH	5.78 ± 0.87	5.62 ± 1.09	.291
Transverse diameter of the LH	4.87 ± 0.68	4.74 ± 0.88	.331
Urethral length	3.91 ± 0.52	4.41 ± 0.71	.471
Urethral sphincter thickness	1.26 ± 0.19	1.34 ± 0.77	.368
SWE mean (Kpa)	39.22 ± 5.83	49.74 ± 8.73	<.001
SWE max (Kpa)	77.11 ± 31.09	96.76 ± 32.2	.001
SWE min (Kpa)	16.62 ± 8.31	14.52 ± 8.42	.174

SWE highlighted significant differences in urethral sphincter elasticity between the 2 groups. The average *Young's modulus* of the urethral sphincter in the SUI group (39.22 ± 5.83 kPa) and the maximum *Young's modulus* (77.11 ± 31.09 kPa) was significantly lower than those in the control group (both *p* < .001), indicating a marked reduction in urethral sphincter stiffness in the SUI group. No significant differences were observed in the minimum *Young's modulus* values (*p* = .174), suggesting that the elasticity under minimal stress was similar between the 2 groups (Table 2).

**Figure 3:**
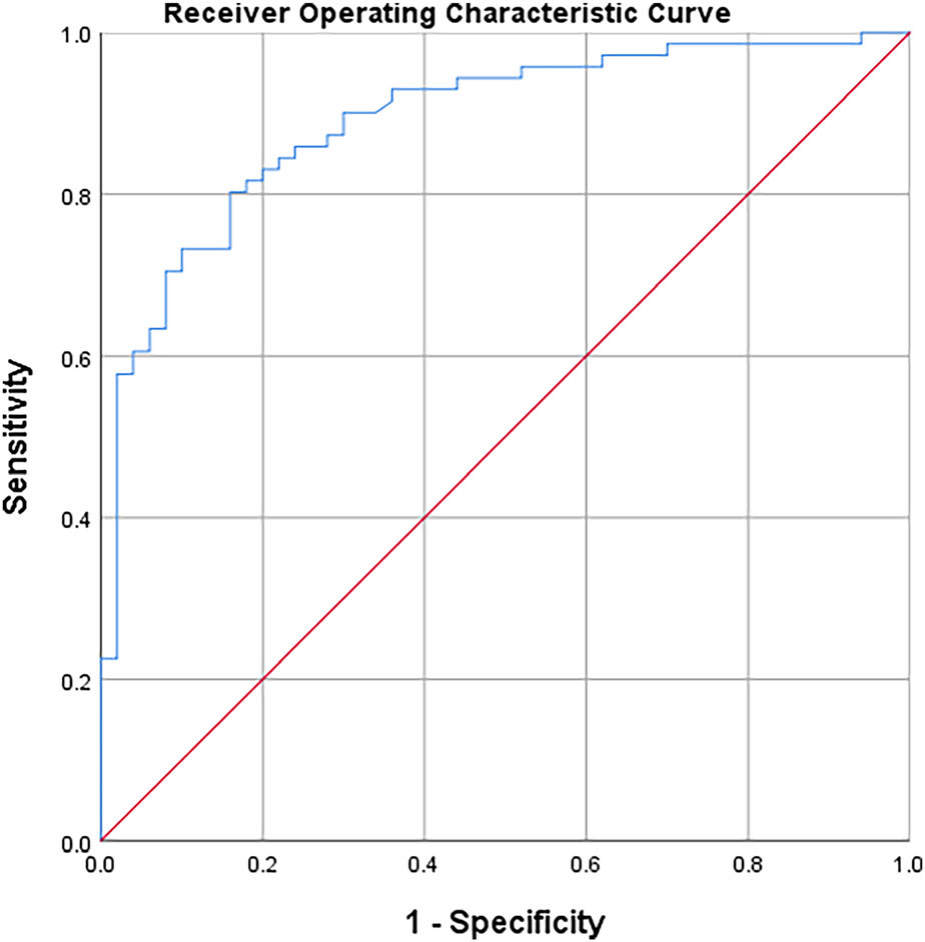
ROC curves for the identification of urinary incontinence. In this analysis, we evaluated the variables that were significant in logistic regression. These variables included age and the mean value of the SWE of the urethral sphincter. For this variable, the area under the curve was 0.891 and the *p*‐value was .001, sensitivity 83.3%; specificity 94.9%.

Binary logistic regression analysis was performed using the clinical data and pelvic floor ultrasound parameters with statistically significant differences, as presented in Figure 3. The analysis revealed that age and average urethral sphincter elasticity were independent risk factors for predicting SUI (both *p* < .05). Age was positively correlated with the risk of SUI, with a regression coefficient of 0.047, indicating that the risk of SUI increases with age. Conversely, average urethral sphincter elasticity was negatively correlated with the risk of SUI, with a regression coefficient of −0.223, suggesting that stronger urethral sphincter elasticity is associated with a lower risk of SUI. Each unit increase in the average *Young's modulus* of the urethral sphincter decreased the likelihood of SUI by 20%. The effect of urethral elasticity on the likelihood of SUI was highly significant (*p* < .000), with individuals with reduced urethral elasticity being more prone to developing SUI.

**Figure 4:**
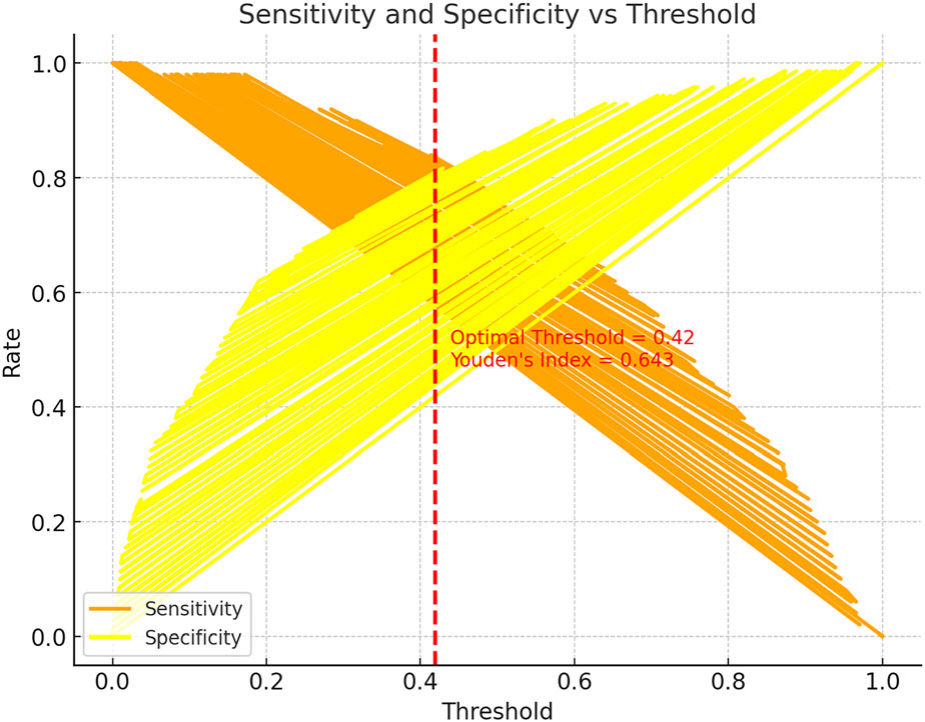
This chart shows how the model's sensitivity and specificity change with different thresholds. The optimal threshold is 0.42, with sensitivity at 0.84, specificity at 0.80, and the Youden index at 0.643. This demonstrates the model's flexibility in adjusting thresholds for different clinical needs.

**Figure 5:**
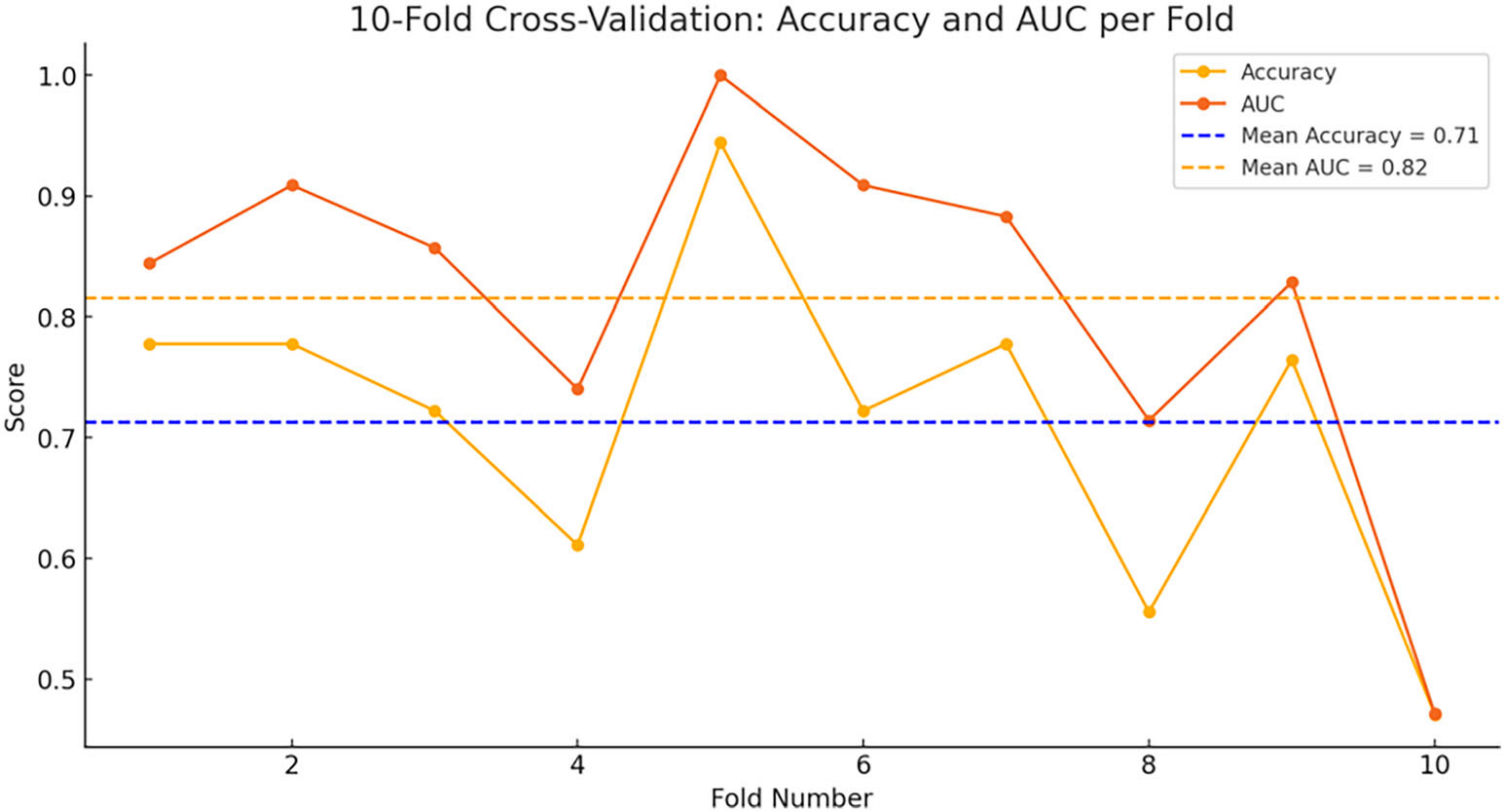
In the 10‐fold cross‐validation, the model achieved an average accuracy of 71.24% and an AUC of 0.816, indicating that the model has a relatively strong ability to distinguish between SUI and non‐SUI, and demonstrates good prediction performance. The standard deviations of the accuracy and AUC were 12.76 and 13.90%, respectively.

The ROC curve analysis of the model revealed an area under the curve (AUC) of 0.891, reflecting excellent discriminatory power and outstanding predictive performance. At a threshold of 0.37, the model achieved an optimal sensitivity of 84% and a specificity of 80%, underscoring the model's effectiveness in distinguishing between SUI patients and healthy controls (Figure 4). These values suggest that the model holds potential for guiding clinical decision‐making in early detection and intervention strategies for SUI. Furthermore, 10‐fold cross‐validation demonstrated stable performance across most folds, with only slight variability in a few folds (Figure 5). Overall, the validation results indicated that the model exhibits good generalizability across different data distributions.

## Discussion

The urethra, a complex multi‐layered structure, consists primarily of the mucosal layer, smooth muscle layer, outer membrane, and the sphincter muscle.^6^ The pathophysiological mechanisms underlying SUI are multifactorial, involving dysfunction at various levels of the urinary tract. While a variety of diagnostic methods for SUI are currently available in clinical practice, each has inherent limitations and drawbacks. Therefore, this study aims to provide a new approach and more accurate means of diagnosing SUI by combining SWE and transperineal pelvic floor ultrasound.

This study reveals that SUI pathogenesis hinges on 2 interrelated yet distinct mechanisms: structural hypermobility (elevated bladder neck mobility) and functional insufficiency (reduced urethral stiffness). The SUI group exhibited significantly lower urethral stiffness (mean *Young's modulus*: 39.22 ± 5.83 kPa versus 52.11 ± 9.24 kPa; *p* < .001) and greater bladder neck mobility (*p* = .034) compared to controls. These findings align with the “2‐hit” model of SUI, where anatomical laxity and sphincter degeneration synergistically impair continence.^11^ These differences were statistically significant (*p* < .05), consistent with previous studies.^7^, ^12^ The observed reduction in urethral stiffness likely stems from age‐ and parity‐related degenerative cascades. Postmenopausal estrogen deficiency accelerates collagen degradation in the urethral extracellular matrix, compromising tensile strength and elastin recoil.^11^, ^13^ Concurrently, vaginal delivery‐induced mechanical trauma—such as pelvic floor avulsion or pudendal nerve injury—exacerbates pelvic floor laxity, destabilizing urethral support.^9^, ^14^ This model explains why multiparous, postmenopausal women face the highest SUI risk. Additionally, the SUI group had a higher number of vaginal deliveries compared to the control group. During childbirth, the compression of the fetal head through the birth canal, along with pressure exerted on the pelvic floor muscles, can cause tearing and stretching of the pelvic floor fascia, leading to ischemia of certain pelvic tissues.^9^ This damage can result in irreversible injury to the urethra and bladder neck, subsequently raising the risk of SUI.^11^ We also observed that bladder neck mobility and bladder posterior angle were significantly increased in the SUI group compared to the normal controls, a finding consistent with the work of Tähtinen et al.^14^ Some researchers have suggested that bladder neck mobility is one of the most reliable indicators for diagnosing SUI and assessing its severity.^15^ The bladder neck, connected to the urethra, reflects the mobility of the urethra. Given its crucial role in urination, any decrease in the bladder neck's support function can lead to changes in its relative position, which in turn may compromise the function of the urethral sphincter, thereby increasing the risk of SUI.^16^ These relative positional changes of the bladder neck are quantifiable through TPUS as increased bladder neck mobility and the widening of the bladder posterior angle.^14^ Therefore, bladder neck dysfunction is a key factor in the pathogenesis of SUI. This finding underscores the importance of the bladder neck in the development of SUI and suggests that the bladder neck's supportive function may directly influence the effective performance of the urethral sphincter. These results provide strong evidence for a more comprehensive understanding of the multifactorial pathogenesis of SUI.

While bladder neck mobility is a recognized anatomical marker of SUI,^14^, ^15^ our integration of SWE highlights its functional consequences. Elevated mobility alters the urethrovesical angle, reducing pressure transmission efficiency during stress.^17^ However, even moderate hypermobility becomes clinically consequential when paired with reduced urethral stiffness—a functional deficit undetectable by traditional imaging. This synergy underscores the need for combined anatomical–functional assessments.

In the logistic regression analysis, we identified age and the average SWE of the urethral sphincter as independent risk factors for SUI. Age was found to have a positive correlation with the risk of SUI, whereas the average elasticity of the urethral sphincter showed a negative correlation with SUI risk, indicating that poorer urethral sphincter elasticity is associated with a higher risk of SUI. This finding aligns with previous studies,^11^, ^18^ which suggest that aging negatively impacts the elasticity of pelvic floor muscles and connective tissues, further increasing the risk of SUI. Moreover, changes in the elasticity of the urethral sphincter, particularly those measured using SWE to assess *Young's modulus*, provide a novel biomarker that offers a more refined and quantitative measure for evaluating the risk of SUI. The observed changes may reflect degeneration in both the structure and function of the urethral sphincter, thus further supporting its critical role in the pathophysiology of SUI. Additionally, cross‐validation results further corroborated the model's stability and excellent generalizability. The predictive model developed here (AUC: 0.891) represents a leap forward in SUI diagnostics. By integrating age and SWE‐derived urethral stiffness, it achieves superior accuracy compared to isolated anatomical parameters. Age, a proxy for cumulative degenerative exposure, synergizes with SWE's real‐time functional insights to identify high‐risk asymptomatic women. Nulliparous women with early declines in urethral stiffness could initiate pelvic floor muscle training (PFMT).^19^ Postmenopausal women with combined hypermobility and stiffness deficits may benefit from PFMT combined with vaginal estrogen. Overall, this multi‐factorial predictive model offers a comprehensive assessment of SUI risk, aiding clinicians in the early identification of high‐risk patients and providing a solid theoretical foundation for the development of personalized treatment strategies.

While SWE is widely applied in liver fibrosis and tumor evaluation,^10^ its utility in pelvic floor dysfunction is emerging. Recent studies^18^, ^19^, ^20^ demonstrate SWE's potential in quantifying muscle stiffness, aligning with our findings on urethral sphincter elasticity. Ptaszkowski et al^20^ used SWE to measure the Young's modulus of the puborectalis muscle to evaluate its function, while Quack^21^ employed SWE as an alternative indicator for assessing the mechanical properties of the patellar tendon and quadriceps tendon. Stafford^18^ combined SWE of male pelvic floor muscles with perineal surface electromyography (EMG) and demonstrated that the stiffness of the urethral sphincter correlates with the contraction force of the sphincter. Similarly, this study found that the Young's modulus of the shear wave elasticity of the female urethral sphincter could serve as a physiological quantitative marker reflecting its functional state, and it holds significant predictive value for female SUI. Our findings are consistent with Stafford's conclusions and further validate the potential application of this method in female SUI. Under normal conditions, the urethral sphincter maintains a certain level of elasticity at rest to sustain urethral closure. In our study, the maximum and average Young's modulus values of the urethral sphincter in the SUI group were significantly lower than those in the healthy control group, reflecting insufficient stiffness of the urethral sphincter in the resting state of women with SUI. The reduced stiffness of the urethral sphincter may be associated with the loss of urethral closure pressure, which, especially in the presence of increased intra‐abdominal pressure (eg, during coughing or running), impairs the ability to effectively cope with elevated bladder pressure, thus leading to urinary incontinence.^22^ Wang et al^11^ confirmed the role of SWE in quantifying the stiffness of the urethral striated sphincter and found that a decrease in shear wave elasticity of the urethral striated sphincter was significantly correlated with SUI, which is consistent with the results of this study. Some researchers^17^ have also reported that the stiffness of the urethral sphincter in women with SUI, as measured by SWE, is significantly lower compared to that of normal women, although they suggest that the correlation between urethral sphincter stiffness and SUI may be weak. In this study, we indirectly quantified the function of the urethral sphincter by measuring its Young's modulus using SWE. The lower the Young's modulus of the urethral sphincter, the greater the impairment of its closure function, which further increases the risk of SUI. SWE is a non‐invasive, user‐friendly technique that allows real‐time monitoring of changes in the *Young's modulus* of the urethral sphincter in different states. Compared to traditional urodynamic testing, SWE is associated with higher patient acceptability, as it is non‐invasive and does not require complex preparation. By integrating SWE with other variables, such as age, a multi‐factorial predictive model can provide reliable and accurate diagnostic evidence, which is of significant importance for early screening, prevention, and treatment of SUI. Early screening of individuals with mild SUI or those at high risk can facilitate timely intervention with physical or pharmacological treatments, preventing disease progression and reducing the need for surgical treatment, thereby easing the economic burden on patients.

This study suggests that urethral sphincter SWE is not only an effective tool for assessing urethral health but also a reliable method for early screening and monitoring of SUI. Furthermore, it offers a novel perspective for exploring the pathogenesis of SUI. Through quantitative analysis of urethral sphincter shear wave elasticity, researchers may uncover the intrinsic relationship between changes in sphincter function and the development of SUI. In terms of treatment, improving urethral elasticity or adopting targeted physical therapies may effectively alleviate or prevent the onset of SUI. The deterioration of urethral sphincter elasticity, associated with the loss of elasticity, reduced stiffness, and impaired contractile function, provides a new therapeutic target for the treatment of SUI. Ultimately, this research offers new directions for understanding the pathogenesis and treatment evaluation of SUI.

In this study, we employed a novel approach by combining transrectal dual‐plane probes with perineal ultrasound to assess urethral sphincter function. Unlike conventional TPUS, the transrectal dual‐plane probe bypasses pubic symphysis obstruction, enabling unobstructed visualization of deep urethral structures.^23^ This innovation, combined with SWE's real‐time stiffness quantification, allows simultaneous assessment of urethral length, sphincter thickness, and elasticity—parameters previously evaluated in isolation.^11^ In addition, we quantified the length, thickness, and elasticity of the urethral striated sphincter and conducted a holistic observation of the pelvic floor using perineal ultrasound. This integrated approach is simple, non‐invasive, and provides a clearer and more comprehensive understanding of the urethral anatomical structure. Notably, the linear array probe placed within the rectum^23^ avoids the obstruction of pelvic bone structures, such as the pubic symphysis, which can hinder the assessment of the urethra during conventional perineal ultrasound, thereby improving the imaging of deep urethral structures. The SWE initiated through this method provides unobstructed views, which are crucial for accurate measurement and analysis of the urethra. Furthermore, the transrectal approach eliminates discomfort caused by the compression of the urethra from the perineal probe, providing higher operational stability. This innovative method offers a new avenue for the early diagnosis and prediction of SUI, demonstrating significant advantages over traditional techniques.

However, this study does have certain limitations. The modest sample size may limit the generalizability of our model, particularly for subgroups such as nulliparous or postmenopausal women. Future multicenter studies with larger cohorts are needed to validate these findings. Second, the absence of urodynamic data limits direct correlation between SWE parameters and functional metrics such as MUCP or ALPP. Future multicenter studies with larger cohorts should validate these associations and explore their integration with urodynamic assessments to enhance diagnostic accuracy.

In conclusion, this study establishes a novel multimodal ultrasound protocol that synergizes anatomical and functional assessments for SUI diagnosis. By identifying reduced urethral stiffness and bladder neck hypermobility as key predictors, our approach provides a non‐invasive, clinically actionable tool for early detection and personalized management. Future research should focus on longitudinal validation and therapeutic applications targeting urethral elasticity restoration.
